# Preoperative clear fluids fasting times in children: retrospective analysis of actual times and complications after the implementation of 1-h clear fasting

**DOI:** 10.1186/s44158-024-00149-3

**Published:** 2024-02-13

**Authors:** Zaccaria Ricci, Denise Colosimo, Luca Saccarelli, Mariateresa Pizzo, Elena Schirru, Salvatore Giacalone, Paola Mancinelli, Gabriele Baldini, Paola Serio

**Affiliations:** 1 Anesthesiology Unit, Department of Anesthesia and Critical Care, Meyer Children’s Hospital, IRCCS, Florence, Italy; 2 Section of Anesthesiology and Intensive Care, Department of Health Sciences, University of Florence, Florence, Italy; 3 Neuro-anesthesiology Unit, Department of Anesthesia and Critical Care, Meyer Children’s Hospital, IRCCS, Florence, Italy; 4 Department of Anesthesia and Critical Care, Azienda Ospedaliero-Universitaria Meyer, Viale Pieraccini 24, 50139 Florence, Italy

**Keywords:** Fasting, Pediatric surgery, Clear fluids, Dehydration, General anesthesia, Complications

## Abstract

**Background:**

Preoperative fasting before elective pediatric surgery is a matter of ongoing debate. The objectives of this study were to evaluate the compliance to a recently implemented preoperative fasting protocol (clear fluids until 1 hour from the induction of anesthesia), to identify predictors of prolonged preoperative fasting time, and to determine whether duration of preoperative fasting was associated with adverse outcomes.

**Methods:**

Retrospective single-center study in an operating theater of a tertiary pediatric hospital.

**Results:**

In a 6-month period, 1820 consecutive patients were analyzed. The data collected in the questionnaire reporting the time of last food, milk and/or liquid intake, and eventual reasons for nonadherence was analyzed. Median (interquartile range) preoperative fasting time was 186 (110–345) min. In 502 patients (27.6%), duration of preoperative fasting to clear fluid ranged from 60 to 119 min, whereas in 616 (34%) it was 120–240 min. The reasons for not respecting fasting time rules are mostly related to communication issues or unwillingness by the patients. A significant difference in fasting times was evident between infants and children older than 10 years (188, 105–290 vs. 198, 115–362; *p* = 0.02). Fasting times were significantly shorter in the inpatient group and in the first scheduled patients of the morning. Clear fluids fasting times were significantly longer in patients with hypovolemia complications than in those without, 373 (185–685) vs. 180 (110–330) min (*p* < 0.0001). Longer fasting times to clear fluids, younger age, and scheduled surgery time were independently associated with the odds of experiencing complications.

**Conclusions:**

In this single pediatric center study, median clear fluids fasting time was three times higher (180 min) than those recommended by the preoperative fasting protocol. Compliance to the protocol was observed in approximately 1 out of 4 patients (27.6%). Longer fasting times were associated with an increased risk of complications, which might be due to dehydration and/or hypovolemia.

**Supplementary Information:**

The online version contains supplementary material available at 10.1186/s44158-024-00149-3.

## Introduction

Preoperative fasting before elective surgery aims at minimizing the risk of inhalation of gastric contents during the induction of anesthesia, a rare but potentially severe complication associated with significant morbidity and mortality [1, 2]. Administration of anesthetic agents disrupts the protective airway reflex, thereby increasing the likelihood of regurgitation and aspiration in patients with full stomach. Although the risk of aspiration is a major concern, real-life data from the APRICOT study, encompassing over 30,000 pediatric patients undergoing general anesthesia across 261 European hospitals, reported a very low incidence (0.06–0.13%) [3]. Furthermore, in case aspiration of clear fluids does occur, independently of the specific fasting regimen employed, clinical consequences are generally mild, usually requiring extended postoperative monitoring and, on occasion, short course of antibiotic therapy [1]. However, prolonged preoperative fasting is associated with children distress, alteration of glycemia, acid base and electrolyte balance, volemia, unpleasant feeling of thirst, and parents’ unsatisfaction [4–6]. Consequently, the European Society of Anaesthesiology and Intensive Care has recently recommended to reduce to 1-h preoperative clear fluid fasting time and to encourage patients to proactively drink up to this time [7]. These recommendations should be applied to all pediatric patients, from premature babies to 18-year-old adolescents, and regardless of the presence of comorbidities that might increase the risk of pulmonary aspiration (i.e., reflux, obesity, diabetes, previous gastrointestinal surgery). In November 2022, our institution officially adopted these new recommendations, implementing a preoperative fasting protocol that encouraged patients scheduled for elective surgery to drink clear fluids until 1 h before induction of anesthesia.

Therefore, the objective of this study was to evaluate the impact of the recently implemented protocol on preoperative fasting duration, specifically focusing on clear fluids, and to determine predictors and outcomes associated with preoperative fasting durations.

## Methods

### Study design

This study was a single-center retrospective observational study conducted in a tertiary level pediatric hospital (Meyer Children’s Hospital, Florence, Italy). Local Pediatric Ethics Committee approval was obtained, with the exemption from informed consent due to the design of the study (Comitato Etico Regionale per la Sperimentazione Clinica della Regione Toscana, Sezione: COMITATO ETICO PEDIATRICO). Strengthening the Reporting of Observational Studies in Epidemiology (STROBE) guidelines were applied [8].

In November 2022, the institutional protocol for preoperative fasting has been revised to comply with the most recent guidelines of the European Society of Anesthesia and Intensive Care. The old protocol consisted of fasting times according to the 6-4-2 rule [9] with institutional median times for clear fluids around 250 min. The new protocol proposed the following preoperative fasting times (6-4-3-1 rule): 6 h for solid foods (any type), 4 h for formula milk, cow’s milk, soy beverage, or for a light breakfast (one cup/200 ml of tea or milk or fruit juice + two rusks), 3 h for breast milk, and 1 h for clear liquids (water with/without sugar, tea or coffee without milk, juices without pulp such as apple or white grape juice). In addition, the new protocol recommended a bundle of interventions aiming at preventing unnecessarily long preoperative fasting times. These included improving awareness and knowledge of parents and staff about the importance of minimizing preoperative fasting, communication between the operating room and the ward in the event of an unforeseen delay with the scheduled surgical procedure, and most importantly emphasized the need of maintaining the patient well hydrated with clear fluids until 1 h before induction of anesthesia.

In order to monitor its correct implementation, a preoperative fasting questionnaire filled by nurses before transferring the child from the ward to the operating room was used, reporting anonymized patient’s data, the time of last food, milk and/or liquid intake, and eventual reasons for nonadherence. The same form was then completed in the operating room by the anesthesiologist, who recorded the time of anesthesia induction and predefined adverse events occurred during induction (see Paragraphs 2.2 and 2.3).

The data collected in the questionnaire was analyzed for monitoring quality of care according to institutional policies and for the purpose of the present study. The association between clear fluids fasting duration with adverse events, admission type (outpatient day surgery, surgical inpatients, medical wards), timing of surgery (elective vs urgent surgery), and scheduled surgery time (i.e., first patient of the day vs. the following ones) was assessed, and independent predictors of adverse events were identified.

### Inclusion and exclusion criteria

From January 2023 to June 2023, consecutive patients 0–18 years old (including prematures) scheduled for elective or urgent surgery (defined as non-emergent surgery within 48 h from admission to the emergency department or until patients were considered medically fit) were enrolled. Patients for whom the questionnaire data was incomplete (missing critical information that could not permit to analyze the data for the purpose of the study, such as clear fluids fasting time, anesthesia induction time, or date of birth) were excluded from the analysis. Since questionnaires were anonymous, it was not possible to retrieve missing data from the electronic medical records.

### Data collection

Demographic and clinical variables were all retrieved from the questionnaires and included age, sex, timing of surgery (elective vs urgent), and type of admission inpatients, outpatients, and “other,” including patients coming from the onco-hematology and medical wards and from the intensive care unit). Moreover, time of the last fluid intake, time of anesthesia induction, reason for nonadherence to the recommended 1-h rule, scheduling of surgery (first patient vs following patients of the surgical list), and adverse events during induction of anesthesia (see details below) were also retrieved from the questionnaires.

### Study outcomes

The primary outcome of the study was to assess the duration of preoperative fasting from liquids during a 6-month period following the implementation of the new institutional preoperative fasting protocol. Secondary outcomes included the overall compliance to the preoperative protocol, causes of nonadherence, and the rate of adverse events during the induction of anesthesia.

### Outcome measures

Distribution of fasting from clear fluids was grouped in three predetermined epochs: < 60 min, 120-min intervals (60–119, 120–239, 240–359, 360–479, 480–599, 600–719, 720–839, 840–959), and > 960 min. Patients were considered compliant with the preoperative fasting protocol if clear fluids fasting time was between 60 and 119 min. The upper limit of this time range was chosen considering the old ESA preoperative fasting guidelines [9] recommending clear fluids until 2 h before the induction of anesthesia and being aware that preoperative clear fluids fasting times remain significantly longer than 120 min worldwide [4, 7].

The adverse events were categorized in two predefined risk groups: hypovolemia/dehydration (group A) and regurgitation/aspiration (group B). Group A included the following:

- Electrocardiogram alterations: Bradycardia, with values below the norm for age that required intervention (atropine, external cardiac massage), tachycardia, with values above the norm for age.
- Hypotension: Systolic blood pressure (SBP) < 60 mmHg in newborns, < 70 mmHg in infants, < 70 mmHg + (age in years + 2) in children 1–10 years old, and < 90 mmHg in children > 10 years old
- Difficult peripheral venous access: Need for more than three attempts in patients not classified as “difficult intravenous access” (DIVA), which is defined as failed access attempts with traditional technique, no visible or palpable veins, and personal history of difficult venous access
- Desaturation: SpO2 < 90% for < 30 s or 5% reduction of basal values if basal SpO2 < 90%.

Group B included the following:

- Regurgitation/aspiration: Persistent cough, obstruction, respiratory distress, increased need for oxygen, the presence of gastric contents in the mouth or active vomiting developing at anesthesia induction, and confirmation of pulmonary aspiration
- Suspected laryngospasm after gastroesophageal regurgitation

### Statistical analysis

All continuous variables have been reported as median and interquartile range (IQR) and categorical variables as absolute numbers and percentage. Comparisons between two groups were performed by using the Mann-Whitney test, while to compare three or more groups, the analysis of variance (ANOVA) test with Holm-Šídák’s multiple comparisons post hoc test was applied. The Fisher test has been used to verify associations between categorical variables. To evaluate the associations, Pearson’s test and logistic regression have been applied where necessary. Odds ratio with 95% confidence interval (CI) was reported. Multivariable logistic regression analysis was built to analyze the association between predefined preoperative variables and adverse events. Data were analyzed with GraphPad Prism 9.0 statistical software (GraphPad Software, San Diego, CA, USA).

## Results

**Fig. 1:**
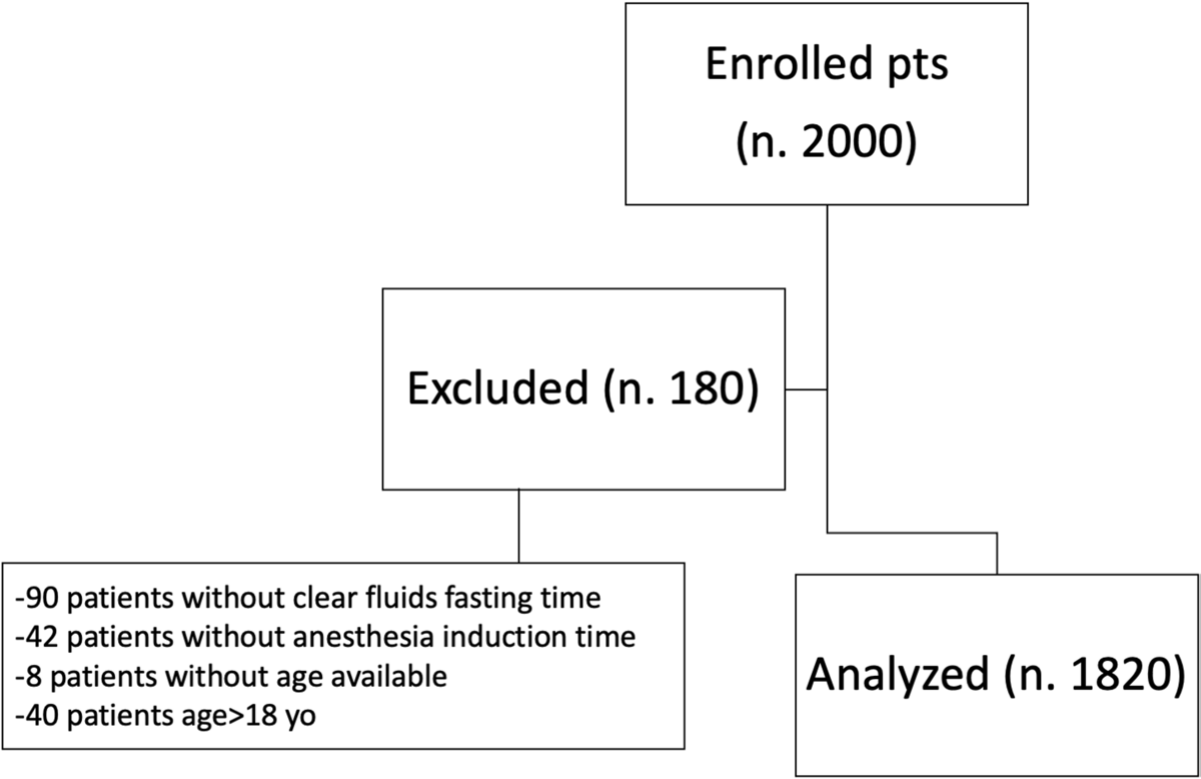
Flowchart of included patients

**Fig. 2:**
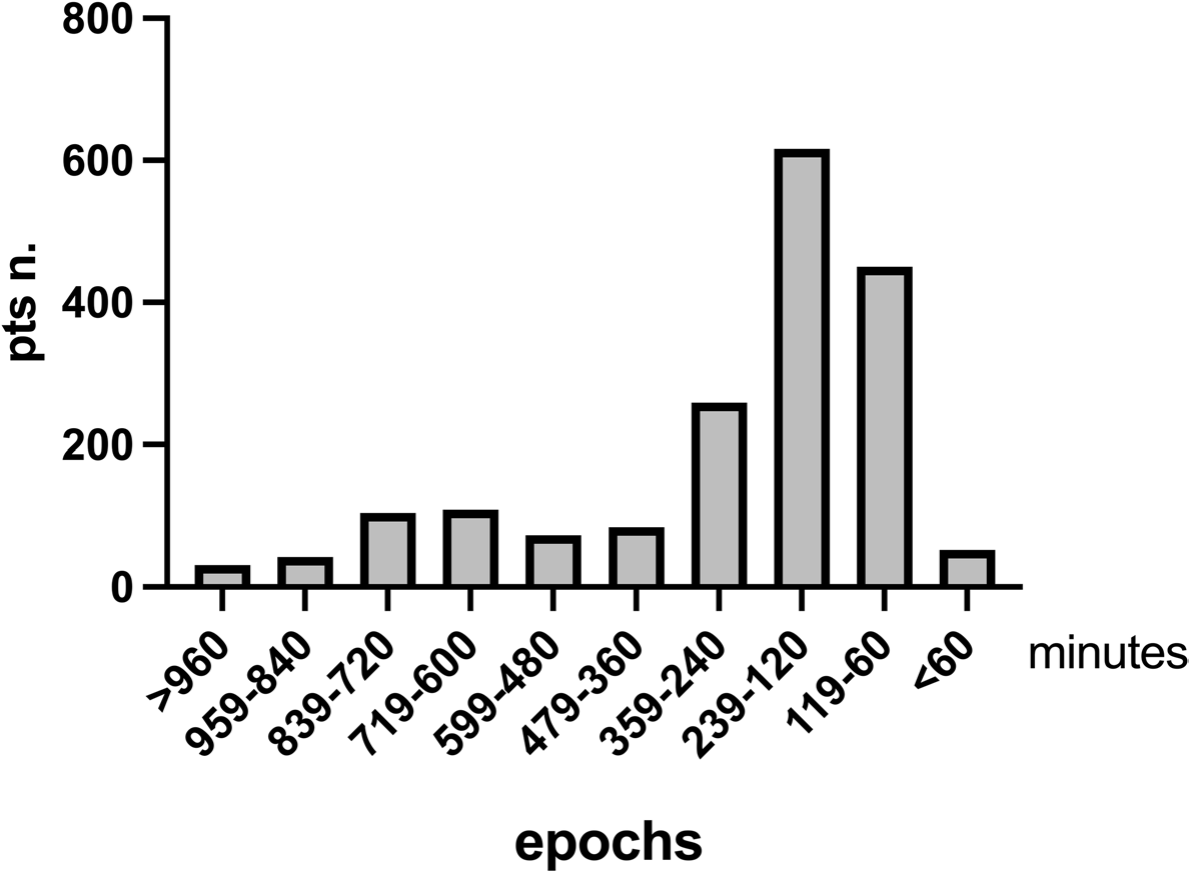
Distribution of clear fluids fasting time, after discretization in time epochs

Overall, 2000 consecutive patients underwent surgery in the analyzed 6-month period, between January and June 2023. Of these, 180 were excluded, resulting in 1820 who have been analyzed (Fig. 1). Demographic characteristics, type of surgery, and preoperative fasting times are described in Table 1. Median clear fluids fasting time was 186 (110–345) min. Distribution of clear fluids fasting times showed that the most frequent fasting duration period was 120–240 min (616 patients, 34%) (Fig. 2). The proportion of patients compliant to the clear fluid fasting protocol (60 to 119 min) was 27.6% (502 patients). According to collected data, 881 times parents or clinicians reported a reason for not respecting the protocol (48% of patients). The reasons for not respecting fasting time rules are reported in Table 2. Fasting times did not seem to change over the analyzed 6-month period (Supplementary Figure 1) and showed to have a weak association with patients age (*r*: 0.05, *p* = 0.04, Supplementary Figure 2). However, after discretization of age classes, a significant difference in fasting times among age classes was shown (ANOVA, *p* = 0.02, Table 1), with the most significant difference between infants and children older than 10 years (post hoc multiple comparison test, *p* = 0.03). When comparing admission types, it appeared that fasting times were significantly longer for outpatients (day surgery) (ANOVA, *p* < 0.0001, Table 1). Patients scheduled as first in the morning showed shorter fasting times than patients scheduled later during the day: 150 (100–531) vs. 195 (120–330) min (*p* = 0.003). There were no differences in preoperative clear fluids fasting time between elective surgery and urgent surgery: 187 (110–360) vs. 180 (110–295) min (*p* = 0.11). Overall, reported adverse events were 98 (5.4%): 95 were in group A (59 difficulty in vein cannulations, 29 electrocardiogram alterations, and 7 hypotension events) and 3 in group B (i.e., 2 episodes of vomiting during anesthesia induction and 1 laryngospasm). The three patients in group B had drunk 88, 110 and 120 min before induction, respectively. Median clear fluid fasting time of patients developing hypovolemia/dehydration adverse events (group A) was significantly longer than those of patients without adverse events (373 (185–685) vs. 180 (110–330) min, respectively, *p* < 0.0001).

**Table 1:** Demographic characteristics and main outcomes

	N°	Fasting from liquids (min)	*p*
All	1820	186 (110–345)	
**Age (years)**			
< 1 (*n*)	171	188 (105–290)	0.02
1–3 (*n*)	219	185 (105–380)	
3–5 (*n*)	232	180 (110–551)	
5–10 (*n*)	560	180 (112–330)	
> 10 (*n*)	638	198 (115–362)	
**Sex**			
Male	879	188 (115–350)	
Female	941	185 (105–330)	
**Timing of surgery**			
Elective	1609	187 (110–360)	0.11
Urgent	211	180 (110–295)	
**Admission type**			
Inpatients	913	150 (100–258)	< 0.0001
Outpatients	799	240 (135–590)	
Others	108	225.5 (151–371)	
**Scheduled as**			
First in the morning	483	150 (100–531)	0.003
Other than first	1337	195 (120–330)

**Table 2:** Reasons of nonadherence to 1-h fasting from liquids rule (881 answers)

Why 1-h fasting for clear liquids rule was not respected?	*n*° (%)
Patient was sleeping	130 (14.7)
Patient refused to drink	344 (39)
Error in communication between staff members or between staff and parents	115 (13)
Organizational issues	18 (2)
Clinical issues	14 (1.6)
Not specified	260 (29.5)

Multivariable logistic regression analysis showed that longer fasting time, younger age, and surgeries not scheduled as first case during the day were independently associated with adverse event (*OR* = 1.002, 95% *CI* 1.001 to 1.003; *OR* = 0.937, 95% *CI* 0.896 to 0.979; *OR* = 0.587, 95% *CI* 0.342 to 0.964, respectively) (Table 3), whereas admission type (inpatients vs. outpatients vs. medical patients) was not (*OR* = 0.713 to 1.680, 95% *CI* 0.713 to 1.680).

**Table 3:** Multivariable regression analysis showing independent associations with the occurrence of any adverse event

Clinical variable	*OR*	95% *CI*	*p*
Fasting time	1.002	1.001 to 1.003	< 0.0001
Inpatients	1.095	0.713 to 1.680	0.676
Age (years)	0.937	0.896 to 0.979	0.004
Scheduled as first case in the morning	0.587	0.342 to 0.964	0.043

## Discussion

Our study contributed to appraise three key messages. First, actual clear fluids fasting times before anesthesia and surgery are still three to four times longer than the time recommended by international guidelines. This finding confirms previous results demonstrating that adherence to a 2-h fasting rule for clear liquids has often resulted in average fasting times ranging from 6 to 13 h [9, 10]. This is likely associated with an ancestral, and not evidence-base, fear of complications by the surgical and anesthesia team. In our study, we were able to achieve a median fasting from clear fluids of about 3 h, with one-fourth of patients below 2 h. Even if accurate institutional average preoperative fasting times before the implementation of this protocol were about 250 min (data limited to a 1-month audit that included only few patients), introducing a 1-h clear fluid fasting rule was perceived as an improvement in managing preoperative fasting by the whole perioperative personnel. As already proposed by other groups, to be consistently compliant to 1-h clear fluids fasting time rule, preoperative recommendations should not restrict clear fluid assumption before induction of anesthesia [11]. Second, the only three adverse events potentially associated with inhalation of gastric content occurred in patients who had had fasted well beyond the recommended time (88 to 120 min), supporting the existing knowledge that the risk of aspiration depends more on the presence of patient or situational risk factors, rather than preoperative fasting time [12]. As a matter of fact, ultrasonographic studies conducted to assess gastric emptying times in healthy pediatric patients have demonstrated average emptying time shorter than previously known, even after a light breakfast [13, 14]. Moreover, the significant association between adverse events and longer fasting times should encourage anesthesiologists, and in general all the perioperative health care personnel, to strictly adhere to the recommended guidelines to avoid excessive fasting times. Hypovolemia and/or dehydration in pediatric patients might lead to clinically significant adverse events [4–6]. Even if clinical consequences of dehydration were only partially and generically verified by our observations, and therefore likely underestimated, our study confirmed the findings reported by other studies [15]. Third, additional independent predictors of adverse events were younger age and surgeries performed late in the day, other than fasting duration. It is known that infants are more frequently subjected to complications with respect to older children [7]. Not surprisingly, it was found that children older than 10 years old fasted significantly longer than infants, although the small difference observed might appear clinically irrelevant (median difference 10 min). Instead, preoperative fasting duration in the other age categories was unexpectedly similar.

It was also found that surgeries scheduled as first case in the morning (8 AM) were independently associated with less adverse events, probably because in our hospital ASA I patients are frequently operated in the morning as day surgery cases. These results should be taken in consideration when planning the operating room schedule, by scheduling high-risk patients earlier in the day and encouraging drinking clear fluids proactively in patients scheduled later. In fact, it might be speculated that, by minimizing preoperative fasting in patients scheduled late, adverse events might be prevented.

To ensure adequate adherence to the preoperative fasting guidelines, anesthesiologists could play a major role, by proactively alerting the ward (or the admission units for day surgery cases) to hydrate the patient as “within 60 minutes the patient will be called.” This behavior may be particularly meaningful for patients scheduled late in the afternoon (or for those patients who have been postponed for emergency surgeries), as their scheduled operating room time may be largely inaccurate and, as a consequence, they may be fasting for excessive time. Our results showed that the reported reasons for not adhering to the implemented protocol included patient/family/caregiver refusal (unwillingness of the children to drink at predetermined times or children who were sleeping were not wakened up to drink) in almost one-third of cases and lack of adequate communication or suboptimal planning (i.e., surgery delays and rescheduling) in another third. It might be possible that a more detailed and resourceful training of patients, families, and caregivers on the importance of minimizing preoperative fasting could further decrease fasting times as recently described in adults [16].

On the other side, it must be emphasized that the 1-h clear fasting rule is not universally accepted as safe rule that ensures an empty stomach at the time of anesthesia induction [17]. Furthermore, to the best of our knowledge, studies that have compared 1-h vs 2-h clear fluid fasting and have eventually demonstrated the superiority of one approach over the other are missing [18]. Moreover, it should be remarked that the certain children might not desire to drink just right before a surgical procedure, and this behavior should be accepted in the absence of strong evidence suggesting benefits and safety of mandatory hydration up to 1 h before surgery. Finally, it should be also considered that the amount of clear fluids necessary to fill the gap of preoperative dehydration before elective surgery (if present) is unknown and questioned if a glass of water 60 min before surgery is actually enough to fulfill this deficit. It is conceivable that the ability to comply with approved protocols and the avoidance of prolonged fasting overnight, or in case of delayed/postponed surgeries, can be more important than focusing on strict rules [19]. For this reason, we considered “compliant” those patients whose fasting times ranged between 60 and 119 min.

### Limitations of the study

This study analyzed a relatively small sample size and for a relatively short period of time, resulting therefore difficult to draw meaningful conclusions about the impact of clear fluids preoperative fasting times on the incidence of adverse events. However, its primary objective was to evaluate the impact of the 6-4-3-1 fasting protocol in the very first months after its application. Previous studies in this field enrolled a significantly larger number of patients [6], which have better determined the association of preoperative fasting time and adverse events. However, despite our relatively small size, sample significant associations between preoperative clear fluids fasting times and adverse events were found. Also, the age distribution of the population studied was not homogeneous and included a higher number of older patients (i.e., > 10 years old). However, this resembled the routine activity of this institution and those of many other pediatric centers in Italy. The adverse events were reported in a questionnaire after occurring and were not instantly measured and/or detected by other personnel. Therefore, it might be possible that the actual clinical burden of the adverse events was higher than reported. However, they were still significantly associated with fasting duration, an important clinical finding of this study. In contrast, the anesthesiologist reporting the adverse events was not blind to the preoperative fasting times, and this might have sensitized the detection of an adverse events in patients with excessive fasting. The lack of accuracy about pre-implementation institutional data did not allow us to verify if preoperative fasting duration improved significantly. However, a rough estimate confirmed a reduction of median times. We did not analyze fasting for solid food and milks, and we could not assess if the new protocol impacted on these outcomes. However, we utilized clear fluids fasting duration as a proxy measure of adherence to the new protocol hypothesizing that median solid food fasting times were proportional to clear fluids fasting times.

## Conclusions

In this single pediatric center, median clear fluids fasting time was three times higher (180 min) than those recommended by the preoperative fasting protocol. Compliance to the protocol was observed in approximately 1 out of 4 patients (27.6%). Longer fasting times were associated with an increased risk of adverse events, which might be due to dehydration and/or hypovolemia.

### Supplementary Information

**Additional file 1.**

## Data Availability

The dataset used and analyzed during the current study is available from the corresponding author on reasonable request.
