# Supplementary material for: Preoperative clear fluids fasting times in children: retrospective analysis of actual times and complications after the implementation of 1-h clear fasting
Source: J Anesth Analg Crit Care. 2024 Feb 13;4:12. doi: 10.1186/s44158-024-00149-3 (PMC10865513; doi:10.1186/s44158-024-00149-3)
Supplement: Supplementary file 1 — Additional file 1. [file 44158_2024_149_MOESM1_ESM.docx]

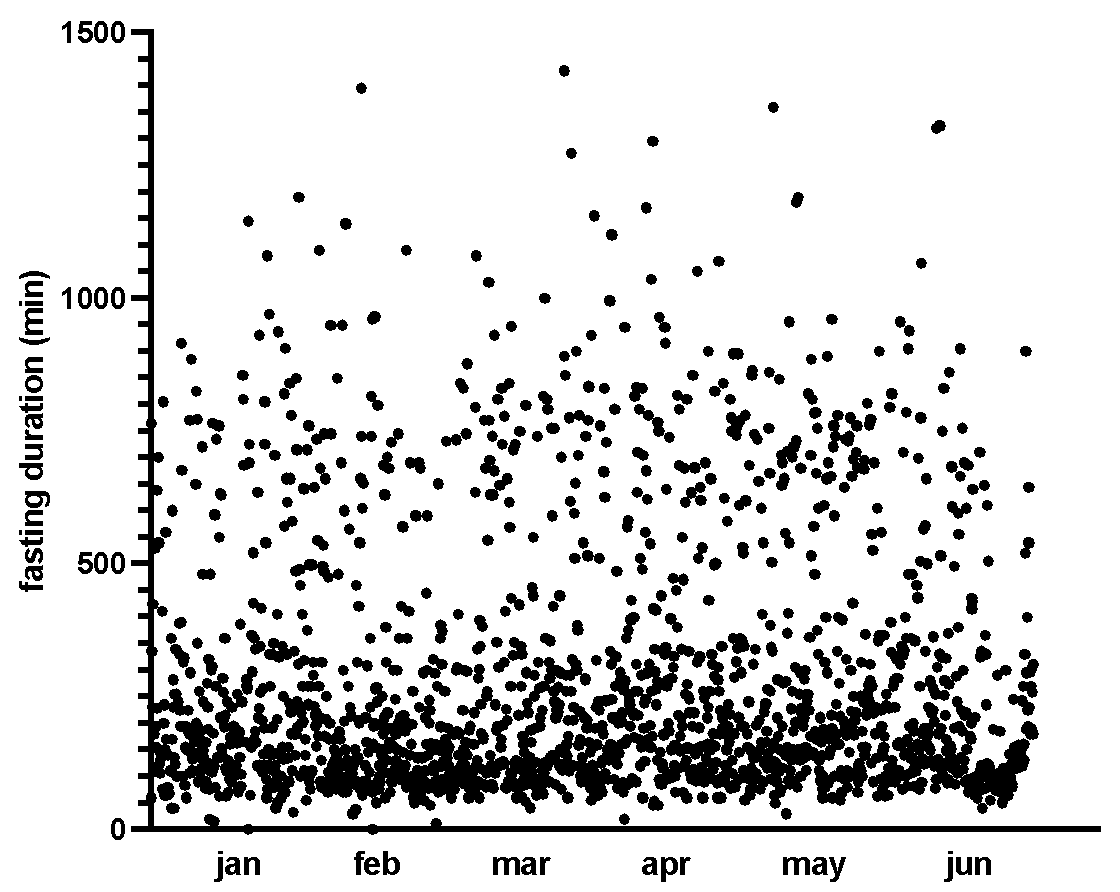


Supplementary figure 1. Scatterplot of clear fluids fasting times in the six analyzed months


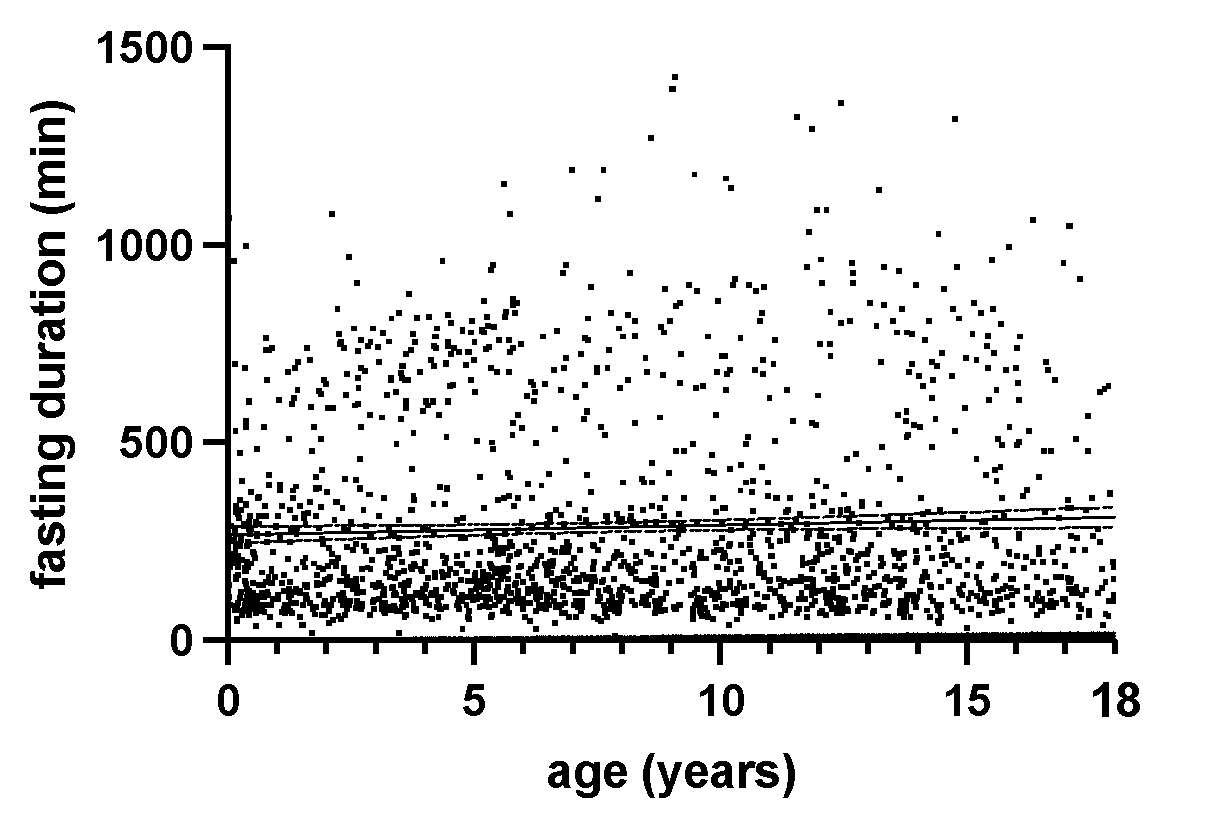


Supplementary figure 2. Regression of age and fasting duration (r:0.05, p=0.04).
